# Obituary for Susan M. MacDonald, M.D.

**DOI:** 10.3390/cells10010087

**Published:** 2021-01-07

**Authors:** Alkis Togias, Marshall Plaut, Jackie Langdon, Ulrich-Axel Bommer, Adam Telerman

**Affiliations:** 1 Allergy, Asthma and Airway Biology Branch, Division of Allergy, Immunology and Transplantation, National Institute of Allergy and Infectious Diseases, National Institutes of Health, Bethesda, MD 20892, USA; mplaut@niaid.nih.gov; 2 School of Medicine, Division of Geriatric Medicine and Gerontology, Johns Hopkins University, Baltimore, MD 21224, USA; jlangdon@jhmi.edu; 3 School of Medicine, Faculty of Science, Medicine & Health, University of Wollongong, Wollongong 2522, Australia; ubommer@uow.edu.au; 4 Institut Gustave Roussy, Unite Inserm U981, 94805 Villejuif, France


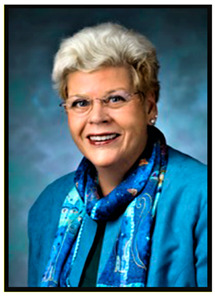


On 9 September 2020, the Allergy and Immunology community lost a prominent member, Susan MacDonald, after a lengthy illness.

After graduating from Regis College and before attending medical school at the University of Massachusetts, Susan worked as a Research Assistant at the laboratory of K. Frank Austen at Harvard Medical School, where she was introduced to immunology. Following medical school, she trained in Internal Medicine at Johns Hopkins Hospital, where she also served as Assistant Chief of Service (Chief Resident) in 1984–1985. Her subspecialty clinical training was in rheumatology, but her research training took place at the Laboratory of Larry Lichtenstein, in the Division of Allergy and Clinical Immunology, Department of Medicine, both at the Johns Hopkins School of Medicine. She went on to join the faculty at Johns Hopkins and attained the rank of Professor in 2004. In 2013, Dr. MacDonald was appointed interim Chief of the Division of Allergy and Clinical Immunology and held this position until her retirement in 2016.

Dr. Susan MacDonald made breakthrough contributions to the understanding of allergic inflammation. She became a world-recognized expert in basophil and mast cell biology, but her major research achievement was the identification and characterization of the biological activity of a key molecule, histamine releasing factor (HRF). In allergic individuals, exposure to allergen immediately triggers a set of symptoms associated with the release of mast cell-derived chemical mediators such as histamine. However, many people also develop a late phase reaction (LPR), occurring anywhere from 2 to 24 h after the initial reaction. This is characterized by a recurrence of symptoms and the release of histamine and other mediators. LPR is induced experimentally when allergic individuals are exposed to the relevant allergen, in the nose, lung and skin. In the natural setting, LPR is probably the mechanism for the persistence of symptoms for hours after exposure to a source of allergen that many patients describe. In cases of systemic anaphylaxis, LPR may explain the biphasic symptomatology that a substantial number of patients develop. Even though the allergen is no longer present in LPR, histamine and other mediators are still released. Thus, another molecule, an HRF, is likely to be responsible for triggering the secretion of the LPR-associated mediators by basophils and/or mast cells. The source and clinical relevance of such mediators have been studied for approximately 40 years. Although several HRFs have been identified, work in the mid-1980s suggested that an IgE-dependent HRF, which interacts with IgE on basophils and mast cells, was a key mediator of LPR [1]. Dr. MacDonald developed a strong research program to isolate, identify and clone this IgE-dependent HRF, and in 1995, she succeeded [2]. HRF functions by interacting with IgE molecules, but HRF works on only certain subsets of IgE molecules (called IgE+) and not on other subsets. It is possible that HRF binds to specific variable regions of IgE and therefore does not bind to all IgE molecules. This may explain why LPR is not a phenomenon that every patient with allergies develops. The importance of HRF in allergic inflammation is not fully understood, but more recent research, especially by Dr. Toshiaki Kawakami and his colleagues, indicates that HRF is critical for inducing allergic inflammation, and that agents that block HRF will block animal models of allergic inflammation [3].

The action of HRF on IgE is an extracellular one, but in her original paper [2], Dr. MacDonald also showed that HRF is identical to an intracellular protein, called translationally controlled tumor protein (TCTP); alas P21, P23, or later fortilin. TCTP was originally discovered in the early 1980s as a highly regulated protein present in proliferating murine cell lines. However, the functional importance of TCTP remained a mystery for a long time, due to the fact that it does not share sequence homology with any other protein family. Hence, Susan’s discovery was of dual importance in the history of the TCTP/HRF proteins—it marks the first description of a functional role of TCTP, and it opened the field for a large number of studies, which investigated the extracellular ‘branch’ of the protein’s function. Meanwhile, also a whole array of intracellular functions has been established, such as cell cycle progression, proliferation, cell survival, as well as its role in the promotion of diseases such as cancer. Most of these functional activities have been reviewed in the first ‘TCTP book’ in 2017, to which Susan MacDonald contributed the chapter ‘History of Histamine-Releasing Factor (HRF)/Translationally Controlled Tumor Protein (TCTP) Including a Potential Therapeutic Target in Asthma and Allergy’ [4]. In that chapter, Susan clearly expresses her fascination with all aspects of TCTP/HRF, including both its intracellular and extracellular functions. You could see this fascination in the brightness of her eyes, like a kid discovering a new world, inviting the scientists, sharing her work and always ready to help. She dedicated all her life to medicine, science and Johns Hopkins. This kind of motivation and enthusiasm has had a lasting impact on her peers. Her colleague, Professor Judith Karp, at the Johns Hopkins Sidney Kimmel Comprehensive Cancer Center, said about Susan: ‘A very talented lady who left an important mark on medicine’.

Known simply as Susan to all who worked with her, she was the first person to turn to when you needed medical advice for a friend or family member. Despite not treating patients for years, her clinical skills were sharp. Dr. Susan MacDonald was a talented, compassionate clinician, and she continued to share these talents with everyone in her circle that needed them.

In 1997, Susan was appointed Deputy Director for Faculty and Career Development at the Johns Hopkins University School of Medicine, Department of Medicine and, in 2002, Associate Chair of Medicine, a position she held for the next 12 years. She was the first woman who held this position at Johns Hopkins. A major responsibility was to oversee the development of more than 575 faculty members and to mentor them through their promotion processes. In this context, she closely mentored more than 35 faculty. Many individuals who Susan mentored remain active and productive members of the scientific community throughout Maryland, and the world. Susan initiated a book that she called “How to Get Promoted at Hopkins” and, in collaboration with the Vice Dean for Faculty Affairs, launched the first edition of the “Silver Book”, which helps the Johns Hopkins faculty in their quest for promotion. Her effort and commitment were further reflected in her appointments at the Johns Hopkins School of Medicine Office of Faculty Development, as an Advisor to the School of Medicine Office of Women in Science and in the School of Medicine Advisory Committee on Mentoring. She was recognized for her extraordinary contributions with several awards, including the Department of Medicine David Levine Excellence in Mentoring Award (2003), the first Vice Dean’s Leadership Award for the Advancement of Women at Johns Hopkins University School of Medicine (2009). From the American Academy of Allergy, Asthma and Immunology (AAAAI), she received the Women Physicians Leadership in Allergy Award (2001), Women’s Involvement Special Recognition Award (2005) and the Gail G. Shapiro Honorary Special Recognition Award (2008). Between 2012 and 2014, she chaired the Leadership Institute and, in 2014–2015, the Task Force on Retention, Inclusion and Diversity of the AAAAI.

Susan MacDonald was also our friend. We spent wonderful moments together and with her beloved husband, Dr. David Herron, and we experienced her warm support in difficult moments. She will be fondly remembered.
